# Understanding Patient Portal Use: Implications for Medication Management

**DOI:** 10.2196/jmir.2589

**Published:** 2013-07-03

**Authors:** Chandra Y Osborn, Lindsay Satterwhite Mayberry, Kenneth A Wallston, Kevin B Johnson, Tom A Elasy

**Affiliations:** ^1^Department of MedicineVanderbilt University Medical CenterNashville, TNUnited States; ^2^Department of Biomedical InformaticsVanderbilt University Medical CenterNashville, TNUnited States; ^3^Diabetes Research & Training CenterCenter for Diabetes Translational ResearchVanderbilt University Medical CenterNashville, TNUnited States; ^4^School of NursingVanderbilt University Medical CenterNashville, TNUnited States; ^5^Department of PediatricsVanderbilt University Medical CenterNashville, TNUnited States

**Keywords:** patient portal, computers, medication adherence, diabetes mellitus, pharmacy, health care disparities

## Abstract

**Background:**

The Internet can be leveraged to provide disease management support, including medication adherence promotion that, when tailored, can effectively improve adherence to medications. The growing adoption of patient portals represents an opportunity to support medication management and adherence more broadly, but virtually no data exist about the real and potential impact of existing portals on these outcomes.

**Objective:**

We sought to (1) understand who uses an existing patient portal and reasons for use and nonuse, (2) understand how portal users are using a portal to manage their medications, and (3) explore participants’ ideas for improving portal functionality for medication management and adherence support.

**Methods:**

A total of 75 adults with type 2 diabetes participated in a mixed-methods study involving focus groups, a survey, and a medical chart review. We used quantitative data to identify differences between portal users and nonusers, and to test the relationship between the frequency of portal use and glycemic control among users. We used qualitative methods to understand how and why participants use a portal and their ideas for improving its medication management functionality.

**Results:**

Of the enrolled participants, 81% (61/75) attended a focus group and/or completed a survey; portal users were more likely than nonusers to participate in that capacity (Fisher exact test; *P*=.01). Users were also more likely than nonusers to be Caucasian/white (Fisher exact test; *P*<.001), have higher incomes (Fisher exact test; *P*=.005), and be privately insured (Fisher exact test; *P*<.001). Users also tended to have more education than nonusers (Mann–Whitney U; *P*=.05), although this relationship was not significant at *P*<.05. Among users, more frequent use of a portal was associated with better A1C (Spearman rho =–0.30; *P*=.02). Reasons for nonuse included not knowing about the portal (n=3), not having access to a computer (n=3), or having a family member serve as an online delegate (n=1). Users reported using the portal to request prescription refills/reauthorizations and to view their medication list, and they were enthusiastic about the idea of added refill reminder functionality. They were also interested in added functionality that could streamline the refill/reauthorization process, alert providers to fill/refill nonadherence, and provide information about medication side effects and interactions.

**Conclusions:**

Although there are disparities in patient portal use, patients use portals to manage their medications, are enthusiastic about further leveraging portals to support medication management and adherence, and those who use a portal more frequently have better glycemic control. However, more features and functionality within a portal platform is needed to maximize medication management and adherence promotion.

## Introduction

Diabetes pharmacotherapy improves glycemic control and prevents diabetes-related complications [1], but many individuals with type 2 diabetes mellitus (T2DM) are nonadherent to prescribed medications [2-4]. Medication nonadherence includes not taking the appropriate dose of a medication or not taking it at the correct time (ie, suboptimal dose adherence), abandoning a medication all together, or not picking up or reauthorizing an existing medication (ie, suboptimal refill adherence) [5]. Optimal diabetes self-care is multifaceted, requiring physical activity, appropriate nutrition, blood glucose monitoring, and medication adherence [6], and yet suboptimal medication adherence alone is independently associated with poor glycemic control [4,7], an increased risk of hospitalizations and mortality [8,9], and higher health care costs [9].

The Internet can provide a platform for disease management support [10], including medication adherence promotion [11,12] that, when tailored, effectively improves adherence to long-term medications [11]. Patient portals are secure Internet-based platforms that offer patients the ability to view their personal health information (PHI), and some portals also allow for 2-way secure messaging between patients and health care providers, and the ability to schedule medical appointments and request prescription refills [13-15]. Emerging evidence suggests portals improve health care quality [16,17] and are associated with favorable patient outcomes [18,19]. For example, among patients with diabetes, using a portal or a comparable system has been associated with performing self-care activities and glycemic control [18,19]. On the basis of this and what we know about the efficacy of Internet-based interventions to promote medication adherence [11], offering medication management support via a patient portal [20] may be an effective means of promoting medication adherence to a large audience over a sustained period of time.

In recent years, patient portal functionality has become more robust. Portals are not only allowing patients to perform the tasks described previously, but they also allow patients to transfer, share, and print PHI [21]; receive generic health information [22] and/or personally relevant health information [22,23]; self-screen for acute health issues (eg, flu) [24]; be coached on how to communicate with providers before medical appointments [25]; and manage medication lists [21,26] among other medication management tasks. From 1 of these studies, we learned that having access to personally relevant health information promotes sustained use of a patient portal [22]. In addition, patients who had access to a medication management module added to an existing patient portal were both willing to use and satisfied with using it to reconcile medication lists and to report side effects, adverse drug events, and issues pertaining to medication nonadherence [20]. However, we know very little about how patients are using standard patient portal functionality for medication management and adherence support purposes and what types of tools are currently absent from these platforms that could be added to meet the medication adherence-related needs of patients.

In an effort to learn how individuals with a chronic illness use patient portal technology to manage their medications, we conducted a mixed-methods study with adults with a T2DM diagnosis who had been prescribed glucose-lowering medications and/or insulin. Our objectives were to (1) understand who uses an existing patient portal and reasons for use and nonuse, (2) understand how portal users are using a portal to manage their medications, and (3) explore participants’ ideas for improving portal functionality for medication management and adherence support.

## Methods

### MyHealthAtVanderbilt

MyHealthAtVanderbilt (MHAV) is a patient portal available to patients receiving care at Vanderbilt University Medical Center (VUMC), a large academic medical center in Nashville, Tennessee. Users of MHAV can manage medical bills, view PHI (eg, vital signs, laboratory results, medication lists, and diagnoses) from their electronic health record (EHR), use secure messaging to communicate with providers and manage medical appointments, access VUMC information (eg, maps, provider information, and telephone directory), use health screening tools to assess symptoms or risk for various conditions [24], view opportunities to participate in research studies, and view educational content linked to the *International Classification of Diseases, Ninth Revision* (*ICD-9*) codes from their EHR [14,23]. There are 2 levels of MHAV access to protect the confidentiality of PHI: upon registering online, patients receive access to certain MHAV functions, but they cannot view PHI until they provide in-person identification at a VUMC clinic [14].

### Participants and Recruitment

To both learn about patients’ experiences with using MHAV to manage their health and medication regimens and get their ideas for leveraging this portal to improve medication management and adherence, we recruited English-speaking adults prescribed medications for T2DM who were patients at VUMC primary care clinics. Recruitment included approaching patients in the clinic waiting room, posting flyers advertising the study, and announcing the study on a VUMC listserv. Interested and eligible patients were identified, and completed informed consent procedures to enroll in the study. All enrolled participants consented to having study personnel review their EHR to collect demographic and clinical information (including medication lists), responded to a question about MHAV use, and were invited to attend a focus group and complete a survey. Enrolled participants who did not attend a focus group were invited to an average of 2.9 (SD 0.5) focus group sessions scheduled at different times of the day. Participants who could not attend a focus group were asked to complete a survey by phone or mail. The Institutional Review Board at Vanderbilt University approved all procedures prior to study enrollment.

### Qualitative Data

We conducted 11 focus groups with 2 to 6 participants each, a trained facilitator (authors CYO or LSM), and a trained note taker who recorded verbal and nonverbal communications. All focus group sessions were recorded and transcribed verbatim, using session notes to link participants’ comments to survey data. Focus group questions pertained to patients’ experiences with diabetes medications, experiences with and attitudes toward using MHAV and other technologies to manage diabetes and medication regimens, and ideas for leveraging MHAV and other technologies to improve medication management and adherence. The primary questions of interest were:

1. Do you use MHAV to manage your diabetes and medication regimens? How/why not?
2. What content, resources, or tools/functions could be added to MHAV to help you manage your medications?
3. What do you think about using MHAV to receive an email reminder when it’s time to refill a prescription or receive text messages when it’s time to take a dose of medication or insulin (ie, dose reminders)? Would these functions be helpful to you?

We stratified sessions by self-reported frequency of patient portal use at enrollment (described subsequently) to homogenize groups relative to the subject matter and elicit different perspectives [27], facilitate a more comfortable discussion about experiences with technology, and understand similarities and differences across types of users rather than to generate thematic saturation within each user group. We conducted 2 focus groups with nonusers, 5 with low users, and 4 with high users. In focus group sessions with nonusers, we asked if participants were aware of the MHAV patient portal. We then showed a video demonstration of MHAV [28], and asked participants if they thought they would want to use MHAV in the future. If not, they were asked why not; if so, they were asked which of the features showcased in the demonstration video would they use and what other features would they like to see added.

### Quantitative Data

#### Demographics

We reviewed the EHR to collect each enrolled participant’s age, gender, and race. Those who responded to a survey also supplied their education, income, and insurance status.

#### Patient Portal Use

At enrollment, we asked participants how often they use MHAV to manage their health on a scale from 1=not at all to 5=very often. We used the response to this question to categorize enrolled participants as portal users or nonusers, and to operationalize users’ frequency of use. For these analyses, those who answered “not at all” were considered nonusers and all others were considered users. We also asked users how long they had used the portal in months and years.

#### Clinical Characteristics

For all enrolled participants, we reviewed the EHR to collect the number and type of prescribed medications and the most recent glycosylated hemoglobin (A1C) value to assess glycemic control. Those who responded to a survey also supplied their duration of diabetes in months and years as well as their height and weight used to calculate body mass index.

### Analyses

All statistical tests were performed using Stata 12 (StataCorp LP, College Station, TX, USA). Descriptive statistics characterized the sample. Mann–Whitney *U* tests and Fisher exact tests examined group differences between those who participated in a focus group and/or completed a survey (n=61) and those who were enrolled only (n=14) on demographics, MHAV use, and A1C, and then between patient portal users (n=62) and nonusers (n=13) on all variables. Next, Mann–Whitney *U* tests and Spearman rho (ρ) correlation coefficients tested the relationships between the frequency of MHAV use and demographics and A1C among MHAV users.

We used NVivo 9 (QSR International, Burlington, MA, USA) to code focus group transcripts. The purpose of our analytic approach was not to reach thematic saturation, but rather to explore participants’ receptiveness to using a patient portal for medication management and adherence, and to generate ideas for how to tailor technologies to meet the needs of patients with T2DM. First, author LSM read transcripts in their entirety, identifying statements pertaining to participants’ opinions about, experiences with, and ideas for using MHAV for medication management and adherence. Units of analysis consisted of statements by single participants and, largely, multiparticipant conversations during which participants built on each other’s ideas, interrupted, offered suggestions, and/or indicated a similar or different experience. Next, authors CYO and LSM iteratively reviewed, integrated, and discussed these data until subthemes emerged.

#### Quality Assurance

We took several steps to enrich the quality of our data and ensure the trustworthiness of our coding process. We participated in, recorded, and analyzed debriefing sessions after each focus group [29]. We stratified focus group sessions by self-reported patient portal use to obtain and compare different perspectives (ie, triangulation of sources) [30], and we used analyst triangulation to explore different interpretations of these data [30].

## Results

We enrolled 75 adults with T2DM with a mean age of 56.9 years (SD 8.8); 67% were female, 63% were Caucasian/white, and 33% were African American/black. See Table 1 for additional summary statistics. Of the enrolled participants, 81% attended a focus group session that included a survey (n=45) or completed a survey by phone/mail (n=16). Nonusers of MHAV were less likely than users of MHAV to participate in a focus group or complete a survey (Fisher exact test; *P*=.01), but there were no differences between focus group/survey participants and enrolled-only participants on age, race, gender, or A1C.

**Table 1:** Participant demographic and clinical characteristics stratified by patient portal use.

Demographics		Patient portal use			
		Nonusers (n=13)	Users (n=62)	Full sample (N=75)	*P* value^a^
Age (years), mean (SD)		58.8 (10.9)	56.5 (8.4)	56.9 (8.8)	.52
**Gender, n (%)**					.20
	Male	2 (15.4)	23 (37.1)	25 (33.3)	
	Female	11 (84.6)	39 (62.9)	50 (66.7)	
**Race, n (%)**					<.001
	Caucasian/white	1 (7.7)	46 (74.2)	47 (62.7)	
	African American/black	11 (84.6)	14 (22.6)	25 (33.3)	
Education^b^ (years), mean (SD)		13.2 (1.8)	15.2 (2.3)	15.0 (2.4)	.05
**Income^b^ (US $), n (%)**					.005
	≤39,999	6 (85.7)	12 (23.5)	18 (31.6)	
	40,000-59,999	0 (0.0)	15 (29.4)	15 (26.3)	
	≥60,000	1 (14.3)	24 (47.1)	24 (42.1)	
**Insurance status^b^, n (%)**					<.001
	Private	1 (14.3)	47 (87.0)	48 (78.7)	
	Public	5 (71.4)	6 (11.1)	11 (18.0)	
	None	1 (14.3)	1 (1.9)	2 (3.3)	
Number of diabetes medications, mean (SD)		1.3 (0.7)	1.3 (0.8)	1.3 (0.8)	.84
**Type of diabetes medications, n (%)**					.56
	Oral agents only	11 (84.6)	40 (64.5)	51 (69.9)	
	Insulin only	1 (7.7)	8 (12.9)	9 (12.3)	
	Both	1 (7.7)	12 (3.3)	13 (17.8)	
Diabetes duration^b^ (years), mean (SD)		7.8 (7.5)	8.0 (6.0)	8.0 (6.1)	.72
Body mass index^b^, mean (SD)		39.2 (12.7)	34.4 (10.2)	35.0 (10.5)	.35
A1C (%), mean (SD)		7.1 (1.6)	7.2 (1.6)	7.3 (1.6)	.71

^a^We conducted Fisher exact tests for categorical variables and Mann–Whitney *U* tests for continuous variables.

^b^Variable collected by survey (n=61).

### Who Uses MyHealthAtVanderbilt?

Of all 75 participants who were enrolled in the study, 83% (n=62) were patient portal users and reported using MHAV sometimes to often (mean 3.6, SD 1.0) [31]. Most portal users (72%) reported at least 1 year of use. As shown in Table 1, MHAV users were more likely than nonusers to be Caucasian/white, have higher incomes, and be privately insured. MHAV users also tended to have more education than nonusers (Mann-Whitney *U; P*=.05) though this relationship was not significant at *P*<.05. We found no other differences between MHAV users and nonusers. Among MHAV users, frequency of use was unrelated to race or indicators of socioeconomic status (SES; ie, education, income, and insurance status). Although there was no difference in A1C between MHAV users and nonusers, more frequent use of the portal was associated with better A1C (ρ=–0.30, *P*=.02) among users.

### Why Do Some Participants Not Use MyHealthAtVanderbilt?

After we showed nonusers the MHAV demonstration video, 4 of 7 nonusers reported they were interested in using the portal, but either had never heard about it, or had heard about it but did not know what its capabilities were. One of these participants said she would certainly use MHAV if she could use a computer:

If I knew how to use a computer, I would use [MHAV]. Because, well, really I think all of it would be helpful. [Scheduling] the doctor’s appointments, paying the bills—about the medications—[being able to request] different medications if you need to do that, and knowing about your test results.

56-year-old female, African American/black, nonuser

In general, nonusers who were interested in using MHAV reported wanting to use the secure messaging feature to schedule appointments and wanting to view their PHI, such as their laboratory results and medication list.

Of the 3 participants who were not interested in using MHAV, 2 participants did not know how to use a computer and felt they had good systems in place for managing their health and medications, and the third participant reported her husband used MHAV and managed her medications as her online delegate (see [14,31] for more detail on the delegate function of MHAV).

### How Portal Users Use MyHealthAtVanderbilt to Manage Their Medications

Themes and subthemes are illustrated with quotations in the text and in Table 2. In focus groups, users described using MHAV to review their medication list and request prescription reauthorizations. Participants who used MHAV to view their medication list shared the information with other providers and/or pharmacists and used this information to ensure they were taking medications correctly:

When I visited the physician about my injury, I thought she suggested I take 600 mg of Motrin for 2 weeks. However, when I looked at my medication list, it showed 400 mg of Motrin twice a day. It was helpful to see what she said, what we had talked about.

69-year-old female, African American/black, user

Frequently, users reported using MHAV’s secure messaging feature to request prescription reauthorizations. The participants who used secure messaging for this purpose consistently and enthusiastically endorsed it for streamlining the reauthorization process:

I use [MHAV] all the time for my prescriptions. When they start to run out—[when] the refills require authorization—I will shoot off a [secure message] to my doctor’s office and they will call the pharmacist and I just go pick it up. They will send me a [secure message] back saying they have sent the prescription and just to pick it up. I think it is really, really great.

66-year-old female, African American/black, user

### Ideas to Improve MyHealthAtVanderbilt’s Functionality for Medication Management and Adherence Support

Participants were averse to receiving dose reminders (ie, reminders to take a dose of medication) from MHAV. In general, participants saw value in dose reminder functionality for children, adolescents, and older patients, but did not like the idea of receiving dose reminders via short message service (SMS) text messages, email, or a phone call. Many participants said they do not use SMS text messaging or email on their mobile phones and thought a phone call reminder would be too intrusive. Those who did use SMS text messaging thought dose reminders would become burdensome and unnecessary:

You know, if I am driving around, I don’t want to get a text message or email thing on my phone saying that you know, something about my health.

56-year-old male, Caucasian/white, user

Others felt email reminders would become annoying and indicated they would just turn off the reminder without taking the medication either because they did not have the medication with them or they would become accustomed to turning off the reminder:

I pretty much take my medicine. I just don’t take it the way I should, but I don’t know if I would like getting a reminder every day, twice a day, to take my medicine. Too much. I have just—I guess with me working and being at my desk, it’s just too much. As I get these little ding-dong bells that pull up on my email...and it would just be overload.

46-year-old female, Caucasian/white, user

However, participants were enthusiastic about leveraging MHAV to improve medication management and promote adherence through other functionality. We categorized participants’ ideas for improving MHAV’s medication management functionality into 3 categories: (1) electronically linking MHAV with pharmacies, (2) MHAV alerting providers to patients’ fill/refill nonadherence, and (3) using MHAV to help patients understand their medications. Specific ideas are presented under each category.

#### Electronically Linking MyHealthAtVanderbilt With Pharmacies

Although participants were satisfied with using MHAV to request prescription reauthorizations, they thought that linking MHAV to pharmacies would have several advantages. They wanted (1) MHAV to send proactive refill/reauthorization reminders to providers and patients, (2) MHAV to automatically send patient-initiated prescription medication fill/refill requests to pharmacies, and (3) MHAV to allow patients to request refills and/or reauthorizations for multiple medications at once.

Participants thought MHAV should send proactive reauthorization reminders to providers and/or patients:

It would be nice if [MHAV] kept up with when the [last prescription] was actually written and, 9 months from now, sent me an email that said, “Our records indicate that your prescription is ending [on] the 15th of next month, would you like a renewal? And what pharmacy?” and you could actually correspond back. Because, in my mind, that would reduce what the clinicians have to do on a daily basis, from all the patients needing renewals on prescriptions. Plus, it’s going to help me remember and I’m not going to have a lapse in time frame where I’m struggling to try to get my [medications].

42-year-old female, Caucasian/white, user

**Table 2:** Participant comments about MyHealthAtVanderbilt (MHAV) and medication management.

Themes and subthemes		Participant quotes
**How portal users use MHAV to manage their medications**		
	Review medication list	It’s really handy to have all that information there—any time you are going to a different doctor that’s outside of Vanderbilt. Because I’m asthmatic and I have like a lot of medications that I only take when necessary—I have a sheet of medications, a full sheet! So it’s nice because when you go to another doctor that doesn’t have access to pull up your medical history, you can go on MyHealth and print it off. So then I can take it with me when I go to a new doctor and [say], “Here, this is what I’m on.” And sometimes it’s helpful for me because I found that the pharmacy—sometimes the milligrams do not match [what’s in MyHealth]. (45-year-old female, Caucasian/white, user)
	Use secure messaging to request prescription reauthorizations	Well, the advantage of doing the little [weekly] pill containers is that when I see that the bottle is getting almost empty, I still have a week’s worth right there in my pill containers, so then I can MyHealth them right away. Cause I’ll look up and go, “I don’t have any refills left this time,” and I MyHealth them right away and ask them to send a prescription to the pharmacy. (46-year-old male, African American/black, user)
**Ideas to improve MHAV functionality for medication management and adherence support**		
	Send proactive refill/reauthorization reminders to providers and patients	So, I think something system-based, that would notify the doctor that, “Hey, unless something has changed in the medical record, you need to—let’s be proactive and have that 90-day [prescription] ready to be called in or sent in or what have you.” Because right now [my pharmacy] has to go back to the doctor’s office and get that. You know, there is a time lag of a couple of days, but I think if it was proactively done through [MHAV], to where it was notifying the doctor and yet also notifying me...that your prescription is ready to be picked up, I think [it] would be really helpful. (46-year-old male, Hispanic, nonuser)
	Automatically send patient-initiated prescription medication fill/refill requests to pharmacies	So you go into MHAV, “I need this refill.” You submit it, and it goes to the pharmacy...And it happens every time, I’m standing out there and I’ll go [to the pharmacy] at 11 and it’s not ready. They close at like 5:30 and I go back at 4. Oh well they haven’t called it in yet. Well we are going to be out, you know, out of medicine. I gotta have insulin for tomorrow...What happens is MHAV will send you a message and say your pharmacy’s been notified of this refill. [Then] when you [go] to the pharmacy, the pharmacist will argue with you and you’ll be like, “You don’t have the medication?” and sometimes that’s a two to three day deal getting this medication. (42-year-old female, Caucasian/white, user)
	Allow patients to request refills and/or reauthorizations for multiple medications at once	That’s one of the things that MHAV could do different is to have a link to the pharmacy. So you go in [to MHAV], I need this refill, you check it, submit it and it goes to the pharmacy...you go in [to MHAV] and it says renew/refill prescriptions and you check the box, say I need medicine A and B and you just check A and B. Prescriptions are already on file. I hit submit and it automatically goes to the pharmacy. (45-year-old male, Caucasian/white, user)
	Alert providers to patients’ fill/refill nonadherence	I think [linking MHAV and the pharmacy] would help. [If] you get a prescription filled for a 90-day supply of Metformin and then 100 days go by and you haven’t called for your refill then something is up. You’ve either been noncompliant and you have way too many pills left or you’re dead, or you’re in the hospital or something has happened, you know. The pharmacy here doesn’t call me and say, “You know what? It’s been 100 days since you refilled your Metformin.” And I’m thinking, like...I’m sure [another company] would call you to find out, “What’s going on? You haven’t gotten your refill lately.” (58-year-old female ,Caucasian/white, user)
	Help patients understand and manage medication side effects	I should be able to go on MHAV and put in Verapamil and pull it up and it tells me, “This is what Verapamil is, what it’s prescribed for, if you start noticing these symptoms, tell your doctor.” When you change pills, you don’t know what is what because you take [a] whole bunch of pills every day...If there were links [on MHAV] where I could have clicked on that medicine and it said, “These are the common side effects. If you have any of these...” Or tell me, “This cough is going to go away in two weeks,” or, “This cough is never going to go away—call your provider.” (42-year-old female, Caucasian/white, user)
	Help patients understand and avoid medication interactions	If I could go into my record...be able to go in there somehow and see all the meds I’m on and have something say, “These meds all work together great,” or, “By taking these three different kinds of medicines you might have [an interaction].” And right now, that’s not available to me. I don’t know [that] unless I sit down with my doctor. If my other doctor puts something on [my medication list], [it’s important] that somehow we know that all these drugs are okay together. (46-year-old male, Hispanic, nonuser)

Participants described how linking MHAV with pharmacies could prevent delayed communication between providers and pharmacies. Several participants described problems that occurred because of slow or poor communication between a provider’s office and the pharmacy:

The problem I had was, like—with my doctor’s direction I can request a medication [reauthorization through MHAV] and they may message me back and they will say something like—this happened last time—the nurse wrote back, “Meds called into pharmacy.” Now, what am I supposed to think? Meds are called into the pharmacy, right? So, I pick up the phone and I call the pharmacy and say, “Do you have [my prescription] ready?” [They said] “No.” I said “Did my doctor’s office call in the prescription?” [They said] “No...There is no record.” But [the nurse] messaged me and said, “Meds called into the pharmacy.” Her interpretation is that—without telling me this—before she gets off work she is going to call them into the pharmacy. So I could have hopped up and said, “Oh, my meds are ready, let me drive down and get my meds”...So once you push that button [to send a MHAV request for a reauthorization], it’s a chain of events that has to like go to your provider’s [office], then the doctor’s order [has to go] to the pharmacy and [then] getting [the prescription] ready.

46-year-old male, African American/black, user

As a result, participants reported going to pick up their prescriptions and finding out the request had not been sent to the pharmacy or the prescription had not been filled as they had expected. Participants also described going through a separate process for each medication when it was due for a refill or reauthorization rather than being able to request that all of their prescriptions be refilled and/or reauthorized at the same time. Participants described how their medications were started at different times and have different supplies (eg, 30 days vs 90 days) and refill amounts (eg, 1 vs 3 refills) resulting in occasions when 1 medication needs to be refilled or reauthorized, but others cannot be requested. As a result, the process of maintaining long-term medications was laborious for those with multiple medications:

For me—because I am on so many different drugs—they run out at different times and I feel like I am always going to the pharmacy, and I hate that. In fact, last week, it was the end of the month and I have like 5 or 6 that were due. I did go without one of my medications for 3 days, which I knew was going to be okay, just so that I could get like 6 of them filled on the same day, so they would run out 30 days later [at the same time]. That is bothersome to me.

46-year-old female, Caucasian/white, user

There was widespread enthusiasm for adding functionality to MHAV to address these problems. Participants wanted to be able to request refills for several medications at a single time and have MHAV send the information directly to the pharmacy when it was time for the prescriptions to be refilled (Table 2). One participant described how he used MHAV in conjunction with a mail-order prescription refill service. He described how he could order all of his medications at once, regardless of their refill date:

Okay, so you have the pill bar, and you’re filling it and you get it all filled and you look in the bottom [of your bottle] and there’s only 3 pills left. I gotta go online to [the refill website], [pull] up my account, and it’s got a whole list of your medications, you just put a check and it tells you—conceivably you could check all the medicines and it has a column that says, “You cannot be refilled before such and such a date.” So if you put a check there, they’ll hold that inactive and when the date comes it trips out and they send it automatically...It tells you how many refills you’ve got left. So during that time, I know that I can go onto MHAV and just send a brief note to my care provider that says, “This script is going to run out in 3 weeks,” and they can refill it for me. Just a couple of key strokes! And Bam!

58-year-old male, Caucasian/white, user

The other group members responded enthusiastically, and thought MHAV should add similar features and functionality.

Participants described an ideal system that would start with a patient receiving a refill reminder from MHAV, or logging onto MHAV to request multiple medications, and end with a secure message from MHAV telling them their prescription had been filled and was ready to be picked up from their pharmacy. However, they emphasized the importance of having tailored functionality. For example, participants wanted to be able to specify how they are alerted (eg, email, phone call, or SMS text messaging) with refill/reauthorization reminders and where they want their prescriptions filled:

If you could have [MHAV] contact any pharmacy, and set that in there, that would be very helpful.

58 year-old, female, Caucasian/white, user

#### MyHealthAtVanderbilt Alerting Providers to Patients’ Fill/Refill Nonadherence

By linking MHAV with pharmacies, participants thought MHAV could be leveraged to monitor patients’ refill adherence:

Something I just thought about is the pharmacy at Vanderbilt is not tied in with MyHealth, or the EHR. The doctor says, “You need to take this.” But there’s no record you ever got it filled and you’re taking it. [The pharmacies] need to be tied in with [the EHR] so when you pull it up, you can look and see. “This was filled on July 8th for 90 days.” That record is [currently] not there.

45-year-old male, Caucasian/white, user

Participants wanted providers to follow up with them if they were not refilling their medications on time (Table 2).

#### Using MyHealthAtVanderbilt to Help Patients Understand Their Medications

Participants also suggested adding MHAV functionality to (4) help patients understand and manage medication side effects, and (5) help patients understand and avoid medication interactions. Participants often sent a secure message to their providers to ask about a medication side effect [32], but they felt that many of their questions could be quickly and easily answered by adding functionality to MHAV. Participants also suggested that MHAV alert patients to possible interactions between their prescribed medications that may be missed when they have several doctors prescribing medications for different purposes:

A lot of drug interactions and stuff are well known and are not particularly well managed because you have so many different doctors prescribing at different times. It would be nice if that logic were built in [to MHAV] and you had some degree of confidence that the more common interactions and stuff are being watched by someone, or [MHAV would let you know] with a little alert.

35-year-old male, Caucasian/white, user

## Discussion

### Principal Findings

Patient portals represent a technology with the potential to facilitate better care of patients, but virtually no data exist about the real and potential impact of these portals on medication management and adherence. To our knowledge, this is the first study to investigate ways to leverage an existing portal to help patients better manage their medications and, in turn, become more adherent. Users of the MHAV portal (both high and low users) reported using secure messaging to request prescription reauthorizations, and suggested adding portal features and functionality to facilitate medication management and promote adherence. Specifically, participants would like MHAV to be linked with pharmacies to create functionality that (1) alerts providers when long-term prescriptions need to be reauthorized and alerts patients when they need to be refilled, (2) reduces communication problems between providers’ offices and pharmacies, (3) allows patients to request multiple medication refills at once, and (4) alerts providers to patients’ prescription fill and/or refill nonadherence. Finally, participants would like MHAV to (5) deliver medication information (eg, side effects, other drugs to avoid) in an accessible and user-friendly format. They also emphasized the importance of being able to tailor both how they receive alerts and which pharmacy they want to use to fill or refill prescriptions. Taken together, these suggestions illustrate patients’ readiness for additional portal-related features and functionality to support medication management and adherence.

As health care organizations and providers begin to use portals to educate patients about their medications and support them with adherence, there is an increased need for these efforts to be theoretically driven, evidence-based, and patient centered. Behavioral medicine experts have long advocated for the role theory plays in the design and content of health promotion programs [33,34]. The first and most important reason is that programs grounded in empirically derived theories are more effective than those that are not [35]. Second, programs grounded in the theoretical processes that regulate behavior can specify and test the critical assumptions of a program’s components to detect exactly why it worked or failed under certain conditions or with certain populations and how it should be improved [33,36]. Both benefits are essential to developing self-care support tools and content within portals that will successfully reduce the personal, social, and economic burden of medication nonadherence. The participants in our study also saw value in integrating currently disjointed systems (ie, portals with pharmacies) to streamline the refill/reauthorization process and to have providers monitor refill adherence. Thus, future research efforts should investigate both the willingness of pharmacies to integrate with patient portal systems to streamline refill and reauthorization processes and of providers to monitor patients’ adherence-related activities. Finally, implementing a portal or a medication management module within a portal should go hand in hand with monitoring use and evidence of stakeholder satisfaction, patient adherence, cost-effectiveness, and impact on clinical care and outcomes.

We also explored the types of patients who are and who are not using MHAV and, separately, the relationship between the degree of use and glycemic control. We found that the groups who often have suboptimal glycemic control (ie, African Americans/blacks and individuals with lower SES) [37] were also less likely to have ever used the portal. We also found that, among portal users, more frequent use was associated with better glycemic control. A few recent studies have reported disparities in patient portal use [19,38-40], including 2 studies with diabetes patients [19,40]. Although our methodology, sample size, and portal in question differ from those studies, we also found that adults with diabetes who were African American/black [19,40] or who had less education [40] were less likely to have ever used the MHAV portal. Furthermore, we identified income and insurance disparities in ever using MHAV, which contributes new findings to this literature. Shaw and Ferranti [19] also reported better glycemic control among portal users versus nonusers. In our sample, there was no difference in A1C between portal users and nonusers. Most nonusers (62%) had A1C values less than 7.0%, suggesting these participants may be using other tools to manage their medications and diabetes. We did find an association between more frequent use of a portal and better glycemic control among users only. This finding extends our limited knowledge about the impact of portals on diabetes outcomes [18]. Although differences in glycemic control based on portal use versus nonuse may be spurious (ie, due to the effects associated with education, income, access to computers, or another variable), our finding that more frequent portal use was associated with better glycemic control among participants who had accessed a portal suggests there may be benefits of using a portal that are independent of the contributions of other characteristics of portal users. Future research with larger samples should explore the independent relationship between frequency of portal use and clinical outcomes, adjusting for race and SES.

### Limitations

Because we used a mixed-method approach, our quantitative findings are limited by our qualitative sampling procedure. We had limited variability in A1C among nonusers as a whole, and particularly among African American/black nonusers, to be able to tease apart the relationships (or lack thereof) between race, SES, A1C, and portal use versus nonuse. Although portal nonusers appear to be using strategies or tools to achieve optimal glycemic control despite not using a portal, additional research with larger, more diverse samples (ie, in terms of SES and glycemic control) is needed to both replicate this finding and identify what strategies are being used to maintain glycemic control among patients who do not use portals. In addition, we likely oversampled MHAV users by promoting the study over a listserv. All but one of the nonusers were recruited from clinic waiting rooms, whereas MHAV users often contacted us from seeing flyers in clinic waiting rooms or seeing the listserv announcement. Moreover, we found nonusers (most of whom we contacted by phone) were more difficult to reach for scheduling and reminding about focus group sessions than users (most of whom we contacted by email). Second, our cross-sectional design limits our ability to discern causal relationships (eg, we cannot conclude that more MHAV use improves glycemic control). Third, our study presents participants’ perceptions of the frequency of using a patient portal, how they use it, and what added functionality would support medication management and adherence, which may not adequately reflect actual opinions and/or behaviors. Finally, the generalizability of these findings to patient populations with lower SES, other chronic illnesses, and/or those patients using other patient portals is limited. Therefore, we recommend future research explore these issues using different research methodologies with a wide range of patient populations and portal platforms.

### Implications for Meaningful Use

Patient portals are increasingly being used to demonstrate meaningful use under the Medicare and Medicaid EHR Incentive Program, which provides financial incentives to providers and hospitals that demonstrate they are implementing EHRs to meaningfully improve patient care [41]. Meaningful use is demonstrated through the achievement of benchmarks including, but not limited to: maintaining an active and correct medication and medication allergy list, identifying patient-specific educational resources and making those resources available to patients, performing medication reconciliation, automatically tracking medications from order to administration using assistive technologies in conjunction with an electronic medication administration record, and providing evidence of patients’ use and engagement with their PHI [41]. As providers and hospitals leverage portals to achieve these benchmarks, it will be important to monitor and learn from portal users and nonusers, understand reasons for nonuse, and identify how to offer medication management and adherence support within portals that meet the needs of patients while also satisfying meaningful use requirements.

### Conclusion

We found that patients use portals to manage their health, including their medications (eg, messaging doctors to reauthorize long-term medications), and are enthusiastic about further leveraging these systems to support medication management. Although some portals have included functionality to support medication reconciliation [26], reduce adverse drug events, and improve patient-provider communication regarding medications [20,42], more functionality is needed to maximize medication adherence promotion.

## Abbreviations

ADA: American Diabetes Association
EASD: European Association for the Study of Diabetes
EHR: electronic health record
MHAV: MyHealthAtVanderbilt
PHI: personal health information
SES: socioeconomic status
SMS: short message service
T2DM: type 2 diabetes mellitus
TRIAD: Translating Research into Action for Diabetes
UKPDS: UK Prospective Diabetes Study
VUMC: Vanderbilt University Medical Center
